# Supplementary material for: Places of safety? Fear and violence in acute mental health facilities: A large qualitative study of staff and service user perspectives
Source: PLoS One. 2022 May 4;17(5):e0266935. doi: 10.1371/journal.pone.0266935 (PMC9067690; doi:10.1371/journal.pone.0266935)
Supplement: S1 File — (DOCX) [file pone.0266935.s001.docx]

**Mental Health Service User Interview Schedule**

| *General* |
| --- |
| How long have you been in here? |
| Have you been here or any other acute ward before? |
| Can you tell me what was happening for you at the time you were admitted here? |
| Who visits you here? |
| Do you take leave? |
| *Architecture (physical space and sensory aspects)* |
| Where in the ward do you spend most of your time? |
| Thinking about the other rooms in the ward, what do you like/dislike about them? |
| Does the look and layout of ward address cultural needs and cultural safety? |
| How do the spaces make you feel? |
| *Therapeutic environment (recovery, therapy, activities)* |
| What therapies or treatment are available to you?  Which ones do you use? |
| *Social organization (ward rules/regimes, social relations and cultural issues)* |
| How do you feel about the rules in the ward?  What do you like/dislike about them? Or why do you say that? |
| **Do you feel safe in here? What about your belongings?** |
| How do you feel about privacy here? |
| How do you feel about the food or meals in here? |
| How do you feel about your interactions with others in here? |
| *Wrap-up* |
| What do you like most about being here? |
| What do you miss the most about being here? |
| Is there anything you would like to see here that would make you feel more comfortable? |
| If there was anything you could change about the way the ward is designed, what would that be? |
| How would you feel if you had to come back here? |
| What would a state-of-the-art mental health ward look like? |

**Staff Interview Schedule**

| *General* |
| --- |
| What is your current position here? |
| What are your hours of work? |
| How long have you worked here? |
| Have you worked in any other psych wards? |
| What training do you have to work here? |
| *Architecture (physical space and sensory aspects)* |
| Where in the ward do you spend most of your time? |
| Thinking about the other rooms in the ward, what do you like/dislike about them?  Are they fit for purpose? |
| **What happens in a crisis?**   1. Fire/earthquake 2. Violence 3. Self-harm |
| Thinking about suicide and self-harm, are there features of the building or its layout or fittings that are designed to minimise self-harm and suicide attempts? |
| Have any issues arisen related to the design or layout of the ward? |
| How does the layout of the ward impact the therapeutic alliance? |
| Does the look and layout of ward address cultural needs and cultural safety? |
| *Therapeutic environment (recovery, therapy, activities)* |
| What do you see as the fundamental purpose of the acute psychiatric ward?  What is the philosophy of care? |
| How do you know when a patient is ready to leave? |
| How do you feel about the available therapies or treatments? |
| Do you have any comment on the cultural appropriateness of available therapies/treatments? |
| How do you feel about the range of recreational activities available for patients? |
| What cultural issues or needs have arisen from patients or their families? |
| *Social organization (ward rules/regimes, social relations and cultural issues)* |
| How do you feel about the rules in the ward?  What do you like/dislike about them? Or why do you say that? |
| **Do you feel safe in here?** |
| How do you feel about privacy here? |
| How do you feel about the food or meals in here? |
| How do you feel about your interactions with others in here? |
| *Wrap-up* |
| What do you like most about working here? |
| What do you like the least about working here? |
| Is there anything you would like to see here that would make your job easier? Or give you greater job satisfaction? |
| If there was anything you could change about the way the ward is designed, what would that be? |
| What would a state-of-the-art mental health ward look like? |
