# Supplementary material for: Places of safety? Fear and violence in acute mental health facilities: A large qualitative study of staff and service user perspectives
Source: PLoS One. 2022 May 4;17(5):e0266935. doi: 10.1371/journal.pone.0266935 (PMC9067690; doi:10.1371/journal.pone.0266935)
Supplement: S2 File — (DOCX) [file pone.0266935.s002.docx]

# **Code book for *Places of safety? Fear and violence in acute mental health facilities: a large qualitative study of staff and service user perspectives***

## **Perception of violence**

One of the nurses was walking past one of the bathrooms. And this patient was watching and as she walked past … opened the door out, so she smacked into the door. (staff A1)

We get more records of interactions between nurses and patients that are problematic, because they record them in the book. But if incidents between patients are to be recorded, one of the patients will have to report it to a nurse, and if thought to be serious enough to record in the incident recording system. So, staff … might not actually report it. (staff B13)

## **Causes of violence**

### ***Individual service user factors***

Because of their delusional beliefs, or their paranoia, or they believe that that staff member has done something to them, and they’re going to get them back. (staff A3)

Sometimes a patient’s illness will cause them to behave in a way that causes other people to want to hit them. (staff A3)

It’s very dangerous and they [people with psychosis] are also … easily angered because of the pent-up frustration from hearing the voices. (service user D3)

Forensics [the forensic mental health service] wouldn’t take them, you know. So, they’re really violent people. (staff A1)

I remember one guy hit [male staff member] with a chair. Knocked him out too. And he did it simply because he wanted to [go to] jail. (staff A1)

And he said, "I'll do what the hell I want to get what I want, and if that means I assault you I will do, you fucking bitch." (staff A3)

They know I’m top dog everywhere I go, and they respect that – there was one day, I got so mad, I punched all the security guards there. (service users C7-C9)

#### **Extra quotes**

We got one guy that was discharged from prison … and they wanted to bring him here, and we resisted and resisted and resisted, and made no difference. And he came here. (staff A1)

Synthetic cannabis and methamphetamine and the two main ones. They come into this service and still be coming down from methamphetamine, and they’re aggressive. (staff B6)

People who have taken drugs and become psychotic are very, very difficult to reason with, because they’re on a completely different planet. (staff B8)

The common symptoms sometimes associated with mental illness is heightened agitation and irritability… they are irritable and agitated towards everyone. (staff C7)

A man was having a complete psychotic break down and threatening staff and patients and picking up chairs and throwing them around. (service user A2)

They remember me because I use to beat them up, I beat them all up. (service users C7-C9)

### ***Built environment***

The closed ward should really have the bigger spaces to separate people. (staff D9)

I can remember this particular time, her talking about assaulting me, where we were in the high needs’ unit … I suddenly thought that nobody in the nursing station can see me. This is quite scary, and my stomach was doing something, and she was looking at me with this look she had. (staff C11)

Doesn’t have an exit door from that [area], which is problematic at times. You can be quite vulnerable if someone’s really aggressive, and you have to retreat to the nurses’ station, and you’ve got nowhere you can go. You’ve just got to hope the door holds. (staff B3)

One person has been locked in HCA [the high care area], a patient, and they got very badly assaulted by [another] patient. (staff B5)

And I’ve been working on shifts where there’s been fights in the corridor, and we’re all looking … you can hear the noise, and there’s like this full-on brawl between two males, … but we didn’t know where it was. (staff B2)

So, there's no air circulating. Too condensed, and it's hot ... and his behaviour started escalating within half an hour of being in that area. ... and then one of the staff was assaulted. (staff A2)

Let us have fucking air. And it’s usually because we’re hyperventilating. We just need to go outside and have cool air, to just calm down. (service user B1)

And that’s not nice to be told that they’re fearful for their safety while you’re standing on the other side of the glass and you’re actually quite terrified yourself. (service user A6)

It’s all shatterproof now because people used to throw chairs through it. (service user A2)

#### **Extra quotes**

You’ll probably find is why you spend a lot of time in the nurses’ station … I feel safe in the nurses’ station. But yeah, I feel safe in the nurses’ station. (staff A1)

The air conditioning; something needs to happen with the air conditioning. It's a real trigger for people. It is extremely hot down the ICU end, as you know. (staff A2)

This was in the open ward and they were waiting for security to turn up, so there’s like 15 nurses and doctors all locked in this fishbowl and there’s all our patients are out here going. (service user A2)

We’ve constantly got clients at the windows. And because it’s not just nursing staff in there, we get a lot of complaints. Like, “People are in there ignoring us.” And I think they’re right when they say that, but it’s not always nursing staff. There’s doctors that aren’t from here, they’re focusing on notes, and they don’t want to look up. So, it then just creates a barrier between us and the clients. (staff A7)

Well, strictly speaking, any patient on … [can’t] function at their best when you're irritable about the heat. Or uncomfortable with the heat. (staff A8)

I know for a fact there was an issue earlier this year where nurses had to retreat. And we were in the nurses’ station, and they had a patient from the ward barricaded in with a chair. And they’re in a room where they had no exit. (staff B3)

[A corridor] is blind-ended. So, it would be easy, particularly at night or after hours, to go down that corridor and have no other staff seeing you, which I think is unsafe. Yeah. You could get trapped in. (staff B12)

So, she was knocked out. And people only knew ‘cause they heard a kafuffle. (staff D3)

### ***Organisational factors***

I know that it was tried a few years ago, before my time, and the assaults went berserk. So even though it’s a non-smoking ward, people do smoke. (staff B9)

Because if these guys can’t smoke, well they take it out on us, basically. (staff A1)

One guy went off his nut the other day before yesterday and started screaming at one of the elderly women because he wanted to go out for a smoke, and they wouldn’t open the door until a certain time. So, he lost it, and threatened her. (service user A3)

So, some staff are reinforcing [the smoke free rules] to the letter and other staff letting it slide. … For example, you'd have a client that is allowed to smoke all day long and then a changeover of shift the nurse that comes on [says], “You know this is a no-smoking area” … before you know it there's a bit of a tiff between … the staff member and the client and then the staff member feels like that the staff member on the earlier shift had set them up for, for all of this. (staff C10)

When there is enough staff, you feel safe. (staff D1)

All the staff are lovely, wonderful, but there needs to be more of them. Because some of them are doing two shifts. (service user A4)

They're completely unqualified and understaffed ... [They need] more staffing, because they can't always…you know, people are waiting. You'll wait there, and wait there, and wait there, until somebody looks at you. (service use A9)

I feel reasonably safe. But then again, I’ve been in these sort of situations for a long time. I know there’s a lot of staff who’ve not long been nursing, or have only got four to five years’ experience, where they still feel scared and upset when an incident occurs. (staff A3)

We’re a 90 per cent workforce female, and 10 per cent male. So, you can imagine the pressure that goes on those female staff, and the pressure that goes on the 10 per cent of males. (staff B3)

You also get the ones that are more likely to take you on, just because they don’t want to hit the small female, but they’ve got no qualms hitting the big guy. … I have been attacked a lot of times. (staff A7)

#### **Extra quotes**

I think that the rules in the ward are good rules, but sometimes when you get clients that don’t want to abide by those rules, you tend to not uphold those rules, because you’re basically too scared. And I’ll admit it, there’s a lot of these patients in this place that intimidate me and make me feel scared. (staff A1)

We had a new psychiatrist turned up here … with all these flash ideas. And he turned up at work one morning and said, “That’s it, no more smoking.” And so, we said, “Oh, okay.” So, I went out to the courtyard and told them all to put out their cigarettes … and one of the nurses got her nose broken. (staff A1)

We’ve got one staff member here, she’s just new, and she’s quite … timid. And these guys just walk all over her something terrible …When I feel like a client's giving her a hard time, then I'll step in ... because you get to know them so well. They become like family in a way, because they're here so often. And they get to know you too. They know you, and they know that "Oh, I can't pull a fast one on her. She's been here too long." So, they seek out the newbies. (staff A1)

One of our clients was using a metal chair to try to get into the courted to smoke at four o’clock in the morning, and I told him that he wasn’t going to. And he then tried to use the chair against me. (staff A3)

I know how to look after myself, so I don’t worry about that. Whereas I know a lot of other people aren’t that, because they don’t have the same abilities, or they aren’t as confident to protect themselves, so they do get scared. And I can’t blame them. (staff A7)

It’s something that I would not enforce. I’m not going to get smacked over because someone wants to go and have a cigarette. (staff B4)

I don't particularly feel unsafe, because of my experience and where I've been, and who I've nursed. But yeah it can be, for some of the new kids on the block, new nurses. Having someone screaming at their face and threatening all this stuff, it can be. (staff B4)

It’s meant to be totally smoke-free. The whole hospital is smoke-free, allegedly, but it’s not. Because I think they trialled it before, and the number of assaults went up. It’s the kind of environment where people are already fairly restricted, so if they are told not to smoke, it can be quite … . (staff B9)

He wanted to go out for a smoke. All he wanted was a smoke. He woke up, I think it was about four or four thirty, I think, he woke up and they wouldn’t let him out for a smoke and he tried to break down the door to go and have a smoke, when it would have been easy just to give him a smoke. (service user A4)

Yeah, they give you a booklet that has all the rules and regulations in it. But It's not adhered to so, it's kind of confusing because you don't really know what's going on. It depends on what staff are on as to what the rules are. (service user A6)

Yeah, I find them quite confusing because they take away ... they took my charger and stuff off people and you have to go to the nurse's station to get it charged. Yet I saw someone sitting in the corridor earlier today plugged into a power point with their charger. (service user A6)

I just feel that the staffing levels, I didn't want to be a burden, here and so, it was quite hard to you know the staff here, I just felt quite under staff and under resourced I didn't really want even put anyone out, so I was quite happy just not really use those [sensory] rooms. (service user C6)

I’ve worked in mental health and I just, I believe that the nurses need more training. (service user A9)

### ***Social milieu***

We’re managing aggressive, aggro males that have used drugs, and have no belief that they’re unwell, with your grandmother. (staff B3)

[I feel unsafe because] the mixing of the males and females and the drug addicts. (service user A2)

This is supposed to be a health facility ... [but] I feel like I'm in prison. (service use A6)

Sometimes, when they just treat us like kids, and we get angry they’ll just be like, “Oh, have some medicine. That’ll make you better.” So, “No, it won’t.” (service user B3)

Because this is not reality. This is like a forced ... you're a child. You're reduced to being three years old. You're getting your nappies changed, sort of shit. It's just real bad. You have to ask for pull-ups, or you have to ask for your period pads and stuff like ... for an independent adult, it's really hard to regress back to a child state, where you're banned from going outside. (service user B1)

There's nothing to do, you get bored, you get agitated. My partner has ADHD and he needs to be doing stuff. And the only programs they have here are for three-year olds. (service user A9)

One person had a fit, a hissy fit, and made a ruckus. But he was pinned down and injected with a drug to calm him down. (service user B6)

#### **Extra quotes**

And, I mean, I know that people have problems and they could probably destroy things or whatever ... I notice that people get very bored. (service user A6)

[Some nurses try] to do the military-style drill tactics when it has been kosher the whole time. So, I suspect a number of TVs are probably going to be broken, you know, due to just complete rebellion. (service user B1)

I can’t do anything. Like all I can do is [pace around] just so not to smash the TVs and shit. (service user B1)

I’m constantly having to deal with people that have no respect for anybody, and are constantly assuming mental superiority over people, including myself. It’s like no one respects me. And I’m tired. (service user B1)

What are they going to do? Cage me? They’ve already done that. So, I’m just going to keep doing what I’m doing. But I’m not liking these doors being closed. (service user B1)

So, I suspect a number of TVs are going to be broken, you know, due to just complete rebellion. We’re grown adults, and we do not need to have bedtimes, or mealtimes, or any time … airtime. Just airtime, going out in the air … this is an awful environment. (service user B1).

### ***Meta-themes***

If you are having a psychotic breakdown like he was, it’s just quite blood rage. You could see in his face and he was just puffed up like a blimmin’ silverback gorilla, basically that’s what he looked like. (service user A2)

When I have had a 90 kg guy throw one of our staff members who is 204 kg against the wall during drug-induced psychosis. That is superhuman strength that we’re dealing with. How am I supposed to restrain that? (staff D2)

# **Consequences of violence**

When you come into work in an HDU [high dependency unit], you accept those things. You know some things are going to happen. At some point during your career you’re going to be on ACC [receiving accident compensation] because you’ve been punched in the face. (staff D2)

I just hate when people are writing policies when they've never even stood foot on an HDU [high dependency unit] ward. (staff D2)

Because [of] the staff safety issues – I think a lot of people have got sick of that and just gone, “You know what, it’s not worth it,” and just, “I’m going to work at a daycare.” (staff D2)

We’re really low on staff and we’ve such high staff turnover, a lot of our extra learning opportunities are getting taken away because we don’t have enough people on the ward to take us off the ward to do for the education. (staff D2)

We have three people with brain injuries off [work] at the moment. (staff D2)

Like, I was actually meant to be on night shift this week, but I had a panic attack and couldn't continue. (staff B2)

I light up and have a cigarette while I'm walking to work. Anxious. (staff A1)

You’ve also got staff that are stressed, so maybe they weren’t calm enough if they need to de-escalate. There’s a lot of reasons, when the staff here are constantly stressed – they don’t get meal breaks, they don’t … you know, they really struggle, and it must be quite hard to sympathise with a person yelling at you. (staff B11)

Sometimes I feel as though they’re going crazy. I feel threatened. (service user B5)

If I go in a psychosis and I’m scared, paranoid and scared, then it makes me even sicker. (service user A8)

It can be the wrong place to be. I’ve got post-traumatic stress, so yeah, a scary situation like that can be really bad. (service user A8)

It’s stigma, yeah, yeah. It feels like they’re working with prisoners. It almost feels like we’re criminals, even though, some of us can function, and others can’t. Different degrees, obviously, on the spectrum but at the end of the day, it just feels like they don’t want to be near you. You know, everything’s blocked off, everything’s, yeah, it’s just, it’s really, really is over the top. (service user A2)

**Extra quotes**

And that’s what makes me really sad, in that I see lots of really good young nurses coming out, and they’re put into really stressful situations fairly early in their practice. And there comes a point, if you’re assaulted, you’re going to really query what you are doing here, and yeah, they don’t stay around. (staff B3)

Not hospitalised assault, so it gets downplayed a little bit, you know. Because it happens all the time. (staff B6)

It gets normalised (in the arena), yeah, for sure. It gets normalised. We always get verbally abused. I just walk past. (staff B6)

It's ridiculous to me. I don't know how you could even make some decisions about my safety when you've never in my shoes. (staff D2)

There’s been two other incidents of nurses being punched quite randomly… and they’ve had to take time off work. (staff D3)
